# Neuronal Porosome Complex: Secretory Machinery at the Nerve Terminal

**DOI:** 10.15190/d.2017.7

**Published:** 2017-07-28

**Authors:** Mzia G. Zhvania, Nino Pochkidze

**Affiliations:** Institute of Chemical Biology, Ilia State University, 3/5 K. Cholokhashvili Avenue, 0162, Tbilisi, Georgia; Department of Brain Ultrastructure and Nanoarchitecture, I. Beriitashvili Center of Experimental BioMedicine, 14, Gotua Street, 0160 Tbilisi, Georgia; Department of Brain Ultrastructure and Nanoarchitecture, I. Beriitashvili Center of Experimental BioMedicine, 14, Gotua Street, 0160 Tbilisi, Georgia.

**Keywords:** Neuronal porosome complex, porosome proteins, synaptic vesicle volume, neurotransmission

## Abstract

Neuronal porosomes are 15 nm cup-shaped lipoprotein secretory machines composed of nearly 30 proteins present at the presynaptic membrane, that have been investigated using multiple imaging modalities, such as electron microscopy, atomic force microscopy, and solution X-ray. Synaptic vesicles transiently dock and fuse at the base of the porosome cup facing the cytosol, by establishing a fusion pore for neurotransmitter release. Studies on the morphology, dynamics, isolation, composition, and reconstitution of the neuronal porosome complex provide a molecular understanding of its structure and function. In the past twenty years, a large body of evidence has accumulated on the involvement of the neuronal porosome proteins in neurotransmission and various neurological disorders. In light of these findings, this review briefly summarizes our current understanding of the neuronal porosome complex, the secretory nanomachine at the nerve terminal.

## 1. Introduction

**Figure 1:**
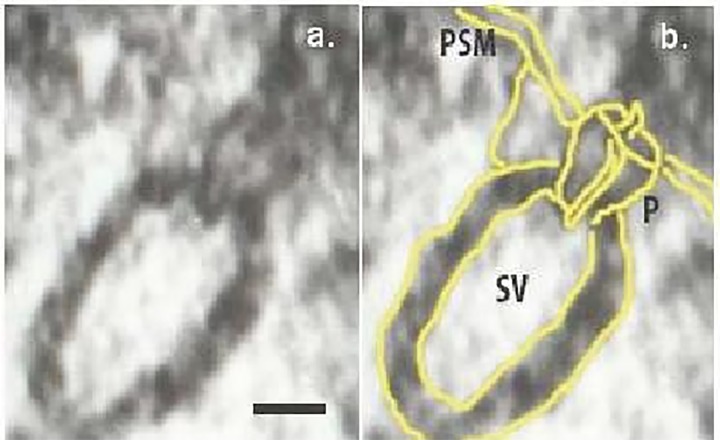
Figure 1. Schematic presentation of strategies for drug repositioning. (a) New indication; an association between a target and a new disease. (b) An association between a drug and a new target. This image was adapted from^11^ with permission. P: porosome; SV: synaptic vesicles; PSM: presynaptic membrane;

Porosomes are cup-shaped secretory nanomachines at the plasma membrane of all cells, including neurons (**Figure 1**), observed using electron microscopy, atomic force microscopy, and solution X-ray, that allow for the precise docking, transient fusion, and fractional release of intravesicular contents from cells^1-12^during secretion. The presence of porosome-like structures hypothesized over twenty-five years ago^13-15^, was first demonstrated to be present nearly two decades ago^1^. One needs to be critically aware regarding the difference between the ‘porosome’ and the ‘fusion pore’. A fusion pore is formed when continuity between two opposing membranes is established. The initial reference of the ‘porosome complex’ as the “fusion pore”^2,3,6^ was a misnomer, since the “fusion pore” is established at the cytosolic face of the cup-shaped porosome complex when membrane-bound secretory vesicles dock and fuse (**Figure 1**). Target SNAREs or t-SNAREs present at the porosome base^2^ and secretory vesicle SNARE or v-SNARE present at the secretory vesicle membrane interact in a rosette pattern^16-21^ to establish the fusion pore in the presence of calcium^22-24^. Viewed from a purely historical perspective, it is of further interest to note, that similar to the hypothesized presence of the porosome^13-15^, following discovery of the SNARE proteins^25-27^ and the establishment of their role in membrane fusion in cells^28^, it was hypothesized that t-SNAREs in the target membrane would interact with v-SNAREs at the secretory vesicle membrane in a rosette or ring configuration^28^, which was physically demonstrated for the first time in a 2002 study^16^ using membrane associated recombinant t- and v-SNAREs^16^. Finally, the observed volume increase in secretory vesicles^29-44^, the molecular mechanism and dynamics of such volume increase in secretory vesicle^45-49^ and its role in cell secretion^50^, have all been determined in the past 30 years, and provide a molecular mechanism of cell secretion.

## 2. Demonstrated role of various neuronal porosome proteins in neurotransmission and neurological disorders

Neuronal porosomes are 15 nm cup-shaped lipoprotein structures composed of nearly 30 proteins^6-8^, compared to a 120-125 nm nuclear pore complex in mammalian cells containing nearly 1000 protein molecules^51^. Neuronal porosomes are secretory nanomachines where synaptic vesicles transiently dock and fuse by establishing a fusion pore for the release of neurotransmitters at the nerve terminal. In the past twenty years, a large body of evidence has accumulated on the involvement of porosome-associated proteins in various neurosecretory diseases^52-76^. For example, the plasma membrane calcium ATPases (PMCA) class of porosome proteins, are known to be involved in maintaining neuronal calcium homeostasis. The PMCA2 class has been shown to co-localize with another porosome protein, synaptohysin^52^. At the presynaptic membrane, Syntaxin-1, also a porosome protein, has been demonstrated to co-localize with PMCA2 and the glycine transporter 2 (GlyT2), that is found coupled to the Na+/ K+ pump, suggesting the presence of a protein complex involved in neurotransmission^53-55^. Studies report that the deletion of PMCA2 generates a phenotype in mice, where the neurons exhibit prolonged hyperpolarized states resulting from an increase in the basal calcium levels^56^. Additionally, mutation in the PMCA2 gene results in homozygous deaf waddler mice (dfw/dfw) with high calcium levels within their synaptic terminals^57^. Similarly, cytoskeletal porosome proteins, such as actin and the alpha chain of tubulin, have been established to be involved in neurotransmission^58^ and various neurological disorders^59^. Latrinculin A, an actin-depolymerizing agent, partially blocks neurotransmitter release from motor neurons^60^. Additionally, actin which is a post-translational product of actin mRNA is important in formation of excitatory synapses, which is promoted by interaction of actin mRNA with the Src-associated in mitosis Sam68 protein. Loss in Sam68 is found to diminish its interaction with actin mRNA leading to lower levels of synaptic actin, leading to neurological disorders involved with abnormal synaptic transmissions^59^. Similarly, although tubulin’s involvement in neurotransmission has not been fully understood, its association with a large group of proteins at the pre-synaptic membrane^61,62^ suggests its critical role in neurotransmission. NAP-22, also known as BASP-1, is a neuronal porosome protein whose involvement in synaptic transmission has been suggested^63-65^. NAP-22 binds to the inner leaflet of lipid rafts suggesting interaction with cholesterol, and it is demonstrated that cholesterol is required to retain the integrity of the neuronal porosome complex^65^. Similarly, the adenylyl cyclase-associated protein-1 or CAP-1 regulates actin polymerization^67^ and both actin and CAP-1 are present in the neuronal porosomal complex^12^. CAP-1 depletion in cells results in lamellopodia growth and F-actin accumulation along with other cytoskeletal abnormalities^68^, reflecting its critical role. Additionally, the porosome protein Na+/ K+ ATPase, plays a critical role in neuronal secretion. Transient blocking of Na+/ K+ ATPase activity by dihydrooubain^69^ results in an increase in both the amplitude and number of action potentials at the nerve terminal^70^. Similarly, changes in SNARE proteins present at the porosome base^2^, are associated with various neurological disorders. SNAP-25 and synaptophysin for example are greatly reduced in neurons of patients with Alzheimer’s disease^71-73^. Furthermore, it is demonstrated that mice that are SNAP-25 (+/-) exhibit disabled learning and memory phenotype, in addition to epileptic like seizures^74^. In contrast, overexpression of SNAP-25 results in cognitive function defects^75^. Studies show that mutations in certain regions of syntaxin 1A, such as the Ca^+2 ^channel-binding region, increases neurotransmitter release, which suggests that syntaxin 1A is involved in regulating Ca^+2^ channel function^76^. Similarly, porosome proteins reticulons contribute to lipid membrane curvature and diseases associated with their deregulation adversely affect neurotransmitter release. These are just a few examples of neuronal porosome proteins that have been implicated both in neurotransmission and in their altered states in neurological disorders.

## 3. Assembly of the membrane-associated neuronal SNARE complex in a rosette or ring conformation to establish the fusion pore at the porosome base

Following discovery of the v-SNARE and t-SNARE proteins^25-27^ and the establishment of their role in membrane fusion in cells^28^, it was hypothesized that both SNAREs in opposing lipid membrane interact in a rosette or ring configuration^28^. This hypothesis was confirmed for the first time in an elegant 2002 study^16^, using membrane associated full length recombinant t- and v-SNAREs and nanometer scale imaging using atomic force microscopy^16^. In a 1998 study^77^, the crystal structure of non-membrane associated truncated t-/v-SNARE complex was solved at 2.4Å resolution. In that research^77^ truncated t- and v-SNAREs, where the hydrophobic membrane-anchoring domain of SNAREs were deleted to overcome solubility problems to generate crystals for X-ray, were used. The atomic force microscopy study^16^ however, soon demonstrated that in absence of membrane association, v-SNARE and t-SNAREs fail to interact and form a rosette or ring, demonstrating the critical role of membrane association on the structure of SNAREs and their interactions. Subsequent studies demonstrate that the size of the SNARE rosette is reflective of the membrane curvature of associated SNAREs^17^. Greater the secretory vesicle size, larger is the size of the SNARE rosette complex^17,20,21^.

## 4. Synaptic vesicle volume regulation in neurotransmission

The requirement of secretory vesicle volume increase in cell secretion^50^ and the molecular mechanism of the process^45-49^, provides for the first time the regulated fractional release of intra-vesicular contents during cell secretion in all cells. The reason and mechanism for the observed volume increase in secretory vesicles in earlier studies^29-44^, has become clear. The presence of adrenergic receptors^49^, heterotrimeric GTP-binding proteins^47,50,78^, ion channels and the water channel aquaporins 1 and 6^78^, confer the capability of synaptic vesicles to finely regulate their volume, hence establish the required intra-vesicular pressure for the release of a precise amount of vesicular content during neurotransmission. Since the importance of lipids both in signaling and membrane protein function has become increasingly clear in the past two decades, not surprisingly, the critical role of cholesterol in synaptic vesicle volume regulation is demonstrated^48^.

## 5. Conclusion

In conclusion, with the discovery of the neuronal porosome complex, and an elucidation of the t-/v-SNARE complex formation and synaptic vesicle volume regulation, a new understanding of neurotransmitter release has come to light, providing a new paradigm in our knowledge of neurotransmitter release. The great body of evidence that has and continues to accumulate since the 1970’s^79,80^ on the fractional or kiss-and-run or kiss-and-release mechanism of neurotransmitter release is clearly explainable with the porosome discovery^81,82^. With elegant secretory nanomachines present in bacteria^83,84^, porosome-mediated secretion in mammalian cells was just waiting to be discovered.
